# Association between glycosylated hemoglobin, diabetes mellitus, and preoperative deep vein thrombosis in patients undergoing total joint arthroplasty: a retrospective study

**DOI:** 10.1186/s13018-022-03328-6

**Published:** 2022-09-29

**Authors:** Xiaojuan Xiong, Ting Li, Bo Cheng

**Affiliations:** 1 Department of Anesthesiology, Army Medical Center of PLA, Daping Hospital, Army Medical University, 10 Changjiang Zhilu, Yuzhong District, Chongqing, 400042 China; 2 Department of Anesthesiology, The First Affiliated Hospital of Chongqing Medical University, 1 Youyi Road, Yuzhong District, Chongqing, 400000 China

**Keywords:** Total joint arthroplasty, Deep vein thrombosis, Preoperative, Diabetes mellitus, Glycosylated hemoglobin

## Abstract

**Background:**

To investigate the association between the level of glycosylated hemoglobin (HbA1c) and preoperative deep vein thrombosis (DVT) and that between diabetes mellitus (DM) and preoperative DVT in patient undergoing total joint arthroplasty (TJA).

**Methods:**

A total of 1386 patients were enrolled. We created the receiver operating characteristic (ROC) curve of HbA1c, and based on the cutoff value, patients were divided into two groups. Risk factors were subsequently examined. Chi-square test or Fisher’s exact test was adopted for enumeration data. The results were expressed in percentages (%), and DVT-related variates were analyzed. We included the variates that were statistically significant in the univariate analysis in the multivariate binary logistic regression analysis and calculated the adjusted odds ratio (OR) and 95% confidence interval (95% CI).

**Results:**

Preoperative DVT was 100 cases (7.22%) and DM in 301 cases (21.7%). We determined the cutoff value of HbA1c of 6.15% using the ROC curve as the area under the curve (AUC) was 0.548. Univariate logistic regression revealed that the risk of preoperative DVT in TJA patients with HbA1c ≥ 6.15%, HbA1c between 7 and 7.9%, HbA1c ≥ 8%, DM, female, and major surgery in the last 12 months increased by 1.84 (*P* = 0.005; 95% CI [1.20–2.80]), 2.22 (*P* = 0.028, 95% CI [1.09–4.52]), 2.47 (*P* = 0.013, 95% CI [1.21–5.04]), 2.03 (*P* = 0.004, 95% CI [1.25–3.30]); 1.85 (*P* = 0.010, 95% CI [1.16–2.95]); and 2.86 times (*P* = 0.006, 95% CI [1.35–6.05]), respectively. And multivariate logistic regression revealed that the risk of preoperative DVT in TJA patients with HbA1c ≥ 6.15%, HbA1c between 7 and 7.9%, HbA1c ≥ 8%, DM patients, female patients, and major surgery in the last 12 months increased by 1.77 (*P* = 0.009, 95% CI [1.16–2.72]); 2.10 (*P* = 0.043, 95% CI [1.02–4.30]); 2.50 (*P* = 0.013, 95% CI [1.22–5.14]); 2.01 (*P* = 0.005, 95% CI [1.23–3.28]); 1.80 (*P* = 0.014, 95% CI [1.13–2.89]); and 3.04 times (*P* = 0.004, 95% CI [1.42–6.49]), respectively.

**Conclusion:**

We conclude that HbA1c ≥ 6.15%, DM, female and major surgery in the last 12 months are the independent risk factors for preoperative DVT in patients undergoing TJA. And patients with a higher HbA1c level are at an increased risk of preoperative DVT.

*Trial registration*: ChiCRT2100054844.

## Background

More TJAs of the hip (THA) and the knee (TKA) are anticipated to be performed in the next decade [1]. VTE, a collective term for DVT and pulmonary embolism (PE), is one of the common complications in patients after TJA and raises perioperative morbidity and mortality [2]. Preoperative DVT can occur in up to 17.9% of TKA patients [3] and up to 29.4% of THA patients [4], respectively. Song K et al. observed that 66.7% of the patients undergoing THA that were diagnosed with postoperative VTE had thrombosis on the same sites as before the surgery [4]. The majority of postoperative DVT is distal venous thrombosis, which accounts for over 90% of all lower extremity DVT, according to research by Deng W et al. [5]. When distal DVT is not treated effectively in time, the probability of distal DVT extending proximally is as high as 20% [6]. Small-sized thrombosis may lead to PE; hence, Smith EB et al. urged attention to medium- and small-sized peripheral DVT in patients undergoing THA [7]. Patients undergoing THA and TKA are at the highest risk of VTE. Approximately 40% to 60% of the patients undergoing major orthopedic surgeries such as TKA and THA develop DVT, and 4% to 10% of the patients without preventive measures experience PE [8, 9]. Therefore, the key to preventing thrombosis in patients undergoing TJA lies in identifying the high-risk factors of preoperative DVT.

According to the latest global figures, 381.8 million adults (8.3%) are affected by DM, and this number is expected to rise to 591.9 million (8.8%) by 2035 [10]. The global population is entering an aging stage, followed by an increasing proportion of patients with DM in TJA [11]. The incidence of DM in patients undergoing THA and TKA is as high as 52% [13]. The overall incidence of postoperative VTE is 46.8% in patients with DM undergoing THA or TKA [5]. HbA1c reflects blood glucose levels over the past two or three months, and the complication rate following TJA has been found to increase linearly with higher HbA1c [14]. A meta-analysis on outcomes of TJA has shown that high HbA1c levels and high perioperative blood glucose levels are associated with a significantly higher risk of periprosthetic joint infection [15]. Lerstad G et al. reported that the risk of VTE increased by 5% per one standard deviation increase in HbA1c [16]. According to Jiao X et al., the incidence of DVT in early stage following TKA is closely associated with DM and is proportional to HbA1c levels [11].

The impact of preoperative DM and elevated HbA1c levels on complications from TJA have all been the focus of most previous studies. Currently, there are no studies to verify the association between the level of glycosylated hemoglobin (HbA1c) and preoperative deep vein thrombosis (DVT), and that between diabetes mellitus (DM) and preoperative DVT in patient undergoing TJA. Therefore, we used data from our hospital to retrospectively analyze their relationships.

## Materials and methods

### Inclusion and exclusion criteria

Inclusion criteria were patients who underwent TJA in our hospital between January 1, 2017, and December 31, 2021, and had HbA1c records before surgery; a total of 1474 TJA patients were enrolled.

Exclusion criteria: (1) a history of VTE (5 cases); (2) use of anticoagulation medications (aspirin, clopidogrel, warfarin, rivaroxaban, dabigatran): atrial fibrillation (10 cases), coronary heart disease (CHD) patients with installed stents and anticoagulant therapy (15 cases); (3) thrombophilic genetic disorders (0 cases); (4) joint infection: knee joint (12 cases), hip joint (5 cases); (5) tuberculosis of the joint: knee joint (6 cases), hip joint (8 cases); (6) tumors of the joints: knee joint (15 cases), hip joint (4 cases); (7) under the age of 20 (8 cases); (8) pregnant (0 cases). Finally, 1387 patients with TJA were enrolled.

### Research method

We created the receiver operating characteristic (ROC) curve of HbA1c, and based on the cutoff value, patients were divided into two groups. The risk factors for DVT before TJA were subsequently examined. Based on their deep vein ultrasound results, patients were divided into two groups: DVT group and non-DVT group. High-risk factors for DVT before TJA were subsequently analyzed. Then, we used multivariate binary logistic regression analysis to verify. This study has been approved by Medical Research and Ethics Review (No. 184, 2022) and registered in the WHO International Clinical Trials Registration (ChiCRT2100054844).

### Data collection

We collected clinical data by accessing the electronic medical records and surgical anesthesia information systems. All patients received pulse Doppler ultrasound with Philips IE33 GE Vivid 9, C5-1 linear probes at a frequency of 5–10 Hz in their lower extremities and were co-diagnosed by two experienced sonographers. The positive criteria for DVT included venous incompressibility, intravascular filling defect, and the absence of a Doppler signal. We also collected DVT formation sites: distal thrombus (thrombi far from the popliteal vein); proximal thrombus (thrombi near the popliteal vein); mixed thrombus (thrombi contained both proximal and distal ends). Patients’ general information included: name, admission number, height, weight, BMI (body mass index), age, gender, and preoperative diagnosis. Their medical records included: preoperative hypertension, DM, CHD, chronic obstructive pulmonary disease (COPD), chronic bronchitis, rheumatoid arthritis (RA), osteoarthritis (OA), cerebral infarction, cancer, renal failure, use of corticosteroids, preoperative smoking, alcohol consumption, major surgery (major surgery, i.e., any surgical procedure that involves anesthesia or respiratory assistance, included cancer and primary THA [17]) in 12 months; laboratory examinations and auxiliary examinations: blood type (type A, B, AB, O), platelet (PLT) count, HbA1c, and the result of preoperative low extremity vein ultrasound.

### Statistical analysis

We used SPSS 26.0 for statistical analyses. Specifically, we determined the cutoff value for HbA1c by establishing the ROC curve for the DVT and HbA1c levels in patients undergoing TJA. The patients were then divided into two groups based on the cutoff value: those with HbA1c levels higher than the cutoff value and those with HbA1c levels lower than the cutoff value. And risk factors were subsequently examined. Chi-square test or Fisher’s exact test was adopted for enumeration data. The results were expressed in percentages (%), and DVT-related variates were analyzed. The variates that were statistically significant in the univariate analysis were included in the multivariate binary logistic regression analysis, and the adjusted odds ratio (OR) and 95% CI were calculated for the evaluation of the association between TJA, DM, HbA1c, and preoperative DVT. *P* < 0.05 was considered statistically significant.

## Results

### General information of study subjects

A total of 1386 patients who underwent TJA in our hospital and had preoperative HbA1c level records were enrolled in this retrospective study. The mean age of all TJA patients was 64.24 ± 0.3 years, 71.24 ± 0.80 years in the DVT group, and 63.7 ± 0.32 years in the non-DVT group (Table 1). Among the 610 TKA cases and 776 THA cases, 516 (37.23%) were male and 870 (62.77%) were female (Table 2); 404 (29.15%) patients had hypertension; 197 (14.21%) patients had preoperative diagnosed DM, and 83 (5.99%) patients had CHD.

**Table 1:** Univariate analysis of preoperative DVT risk in patients undergoing TJA

	DVT (100)	Non-DVT (1286)	*P*
Height (cm)	156.96 ± 0.64	158.33 ± 0.23	0.047
Weight (kg)	60.17 ± 0.89	61.59 ± 0.29	0.130
BMI (kg/m^2^)	24.45 ± 0.36	24.45 ± 0.10	0.777
Age(year)	71.24 ± 0.80	63.7 ± 0.32	0.000
PLT (10^9/L)	226.72 ± 10.19	211.12 ± 2.02	0.051
HbA1c (%)	6.30 ± 0.13	6.03 ± 0.03	0.053

*BMI* Body mass index; *DVT* deep vein thrombosis; *HbA1c* glycosylated hemoglobin; *TJA* total joint arthroplasty

**Table 2:** Univariate analysis of preoperative DVT risk in patients undergoing TJA

Influencing factor	Chi-square test value	*P*
Gender	6.90	0.010
Hypertension	3.22	0.086
CHD	0.775	0.379
COPD	0.68	0.323
Chronic bronchitis	0.08	0.784
Cerebral infarction	1.08	0.302
Major surgery in the last 12 months	8.22	0.010
Cancer	0.47	1.000
Renal failure	0.80	0.362
Depression	0.16	0.693
Corticosteroid	3.73	0.063
Smoking	2.17	0.190
Drinking	1.48	0.295
Blood type	1.76	0.624
DM	8.47	0.007
Classification HbA1c 6.15%	8.11	0.006
HbA1c Level	10.71	0.025

*CHD* Coronary heart disease; *COPD* chronic obstructive pulmonary disease; *DM* diabetes mellitus; *DVT* deep vein thrombosis; *HbA1c* glycosylated hemoglobin; *TJA* total joint arthroplasty

### HbA1c

**Fig. 1:**
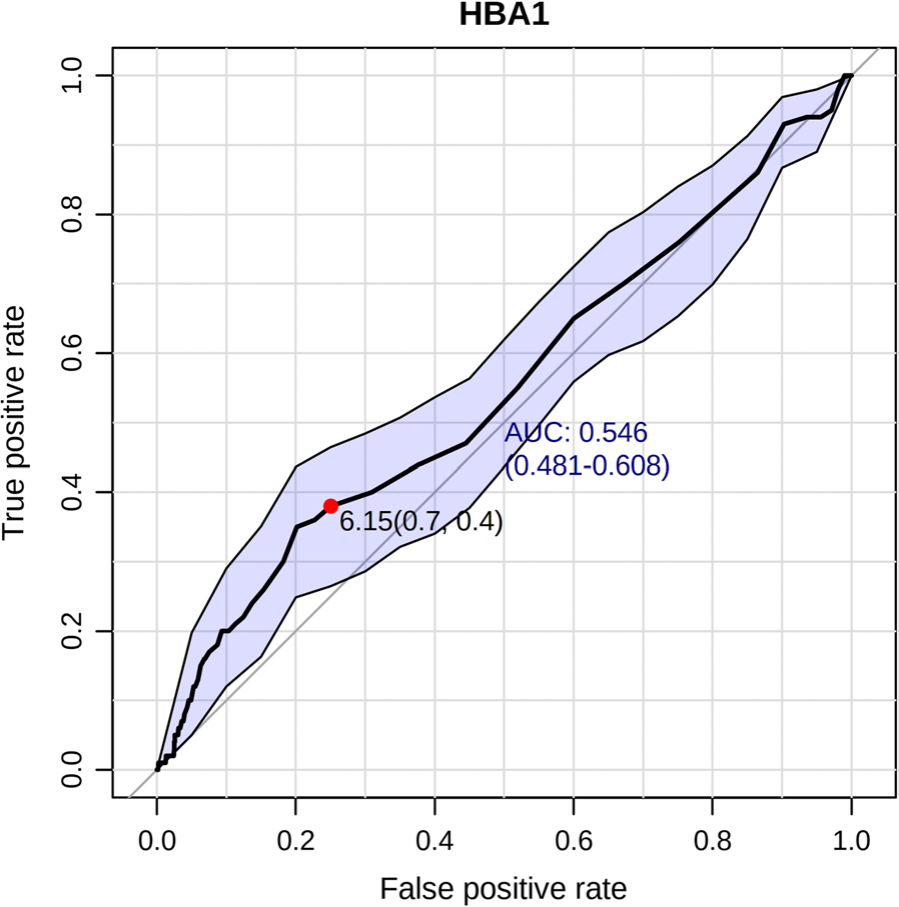
Receiver operating curves of HbA1c for predicting preoperative DVT in patients undergoing TJA. *DVT* Deep vein thrombosis; *HbA1c* glycosylated hemoglobin; *TJA* total joint arthroplasty

We determined the cutoff value of HbA1c of 6.15% using the ROC curve. The AUC was 0.548, *P* = 0.006, 95% CI (0.481–0.608) (Fig. 1). Based on the cutoff value, we divided the patients into two groups: the HbA1c ≥ 6.15% group (380 cases) and the HbA1c < 6.15% group (1006 cases). The basic information of the two groups is shown in Table 3.

**Table 3:** Univariate analysis of classification of HbA1c in patients undergoing TJA

	HbA1c ≥ 6.15%	HbA1c < 6.15%	*P*
Height (cm)	158.13 ± 0.41	158.27 ± 0.25	0.770
Weight (kg)	64.55 ± 0.54	60.42 ± 0.31	0.000
BMI (kg/m2)	25.79 ± 0.18	24.12 ± 0.11	0.000
Age (year)	67.94 ± 0.44	62.95 ± 0.37	0.000
PLT (10^9/L)	213.17 ± 3.42	211.93 ± 2.45	0.787

*BMI* Body mass index; *HbA1c* glycosylated hemoglobin; *PLT* platelets; *TJA* total joint arthroplasty

Diagnosed DM occurred in 197 cases (14.21%). The HbA1c levels of DM patients were 7.83 ± 0.13% and those of non-DM patients were 5.76 ± 0.02% (*P* = 0.000). According to the American Diabetes Association, DM is defined as HbA1c ≥ 6.5% and prediabetes is HbA1c between 5.7 and 6.4% [18]. Therefore, in our study, undiagnosed DM patients were those who were not diagnosed with DM upon admission to the hospital but had HbA1c ≥ 6.5%. 301 patients had DM, including diagnosed and undiagnosed, with a total incidence of 21.7%. Among them, undiagnosed DM patients were 104 cases (34.55%). Patients with prediabetes were 573 cases (41.34%), and it was not found that their incidence of DVT before TJA increased.

### Characteristics of DVT formation

The preoperative DVT in our study included all symptomatic DVT and asymptomatic DVT. Among the 100 cases (7.22%) with DVT before TJA, there were 74 cases (74%) with distal thrombus, 12 cases (12%) with proximal thrombus, and 15 cases (15%) with mixed thrombus. Inferior vena cava filters were used for the proximal and mixed types of thrombus and low molecular weight heparin for the distal thrombus. None of our TJA patients had PE during the perioperative period. Among the 360 patients undergoing TJA with HbA1c ≥ 6.15%, preoperative DVT occurred in 38 cases (10.56%); among the 1026 patients undergoing TJA with HbA1c < 6.15%, preoperative DVT occurred in 62 cases (6.04%). The grade of HbA1c levels and the incidence of preoperative DVT are shown in Table 4.

**Table 4:** The grade of HbA1c and the incidence of preoperative DVT in patients undergoing TJA

	HBA1				
	< 6.15%	6.15–6.9%	7.0–7.9%	≥ 8%	Total
DVT	62/1062 (6.04%)	18/207 (8.70%)	10/80 (12.5%)	10/73 (13.7%)	100/1386 (7.22%)

*HbA1c* glycosylated hemoglobin; *DVT* Deep vein thrombosis; *TJA* Total Joint Arthroplasty

### Analysis on high-risk factors of preoperative DVT in patients undergoing TJA

**Fig. 2:**
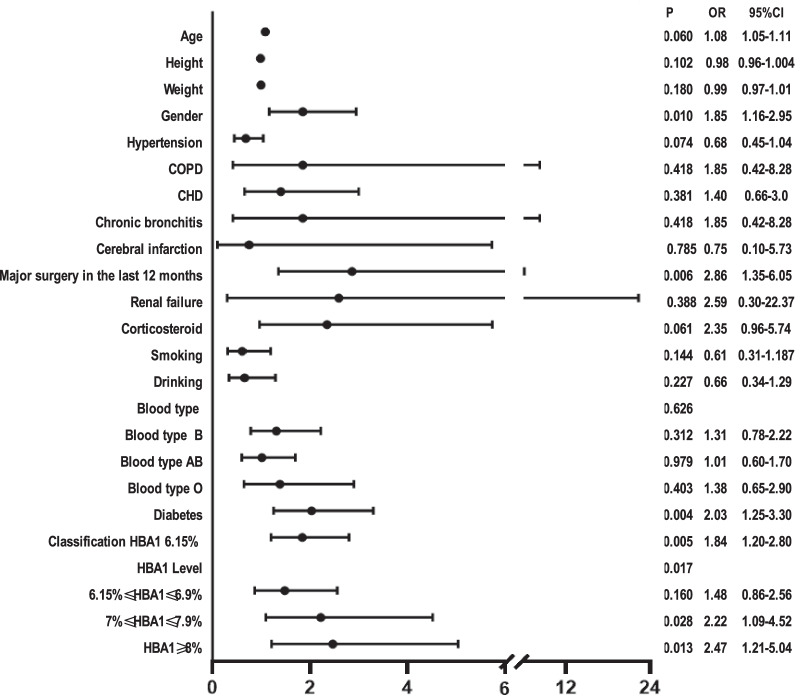
Univariate logistic regression analysis of high-risk factors of preoperative DVT in patients undergoing TJA. *CHD* Coronary heart disease; *COPD* chronic obstructive pulmonary disease; *DM* diabetes mellitus; *DVT* deep vein thrombosis; *HbA1c* glycosylated hemoglobin; *TJA* total joint arthroplasty

Univariate logistic regression revealed that the risk of preoperative DVT in patients undergoing TJA with HbA1c ≥ 6.15%, HbA1c between 7 and 7.9%, HbA1c ≥ 8%, DM patients, female patients, and patients who underwent major surgery in the last 12 months increased by 1.84 (*P* = 0.005; 95%CI [1.20–2.80]), 2.22 (*P* = 0.028, 95% CI [1.09–4.52]), 2.47 (*P* = 0.013, 95% CI [1.21–5.04]), 2.03 (*P* = 0.004, 95% CI [1.25–3.30]); 1.85 (*P* = 0.010, 95% CI [1.16–2.95]); and 2.86 times (*P* = 0.006, 95% CI [1.35–6.05]), respectively (Fig. 2).

**Fig. 3:**
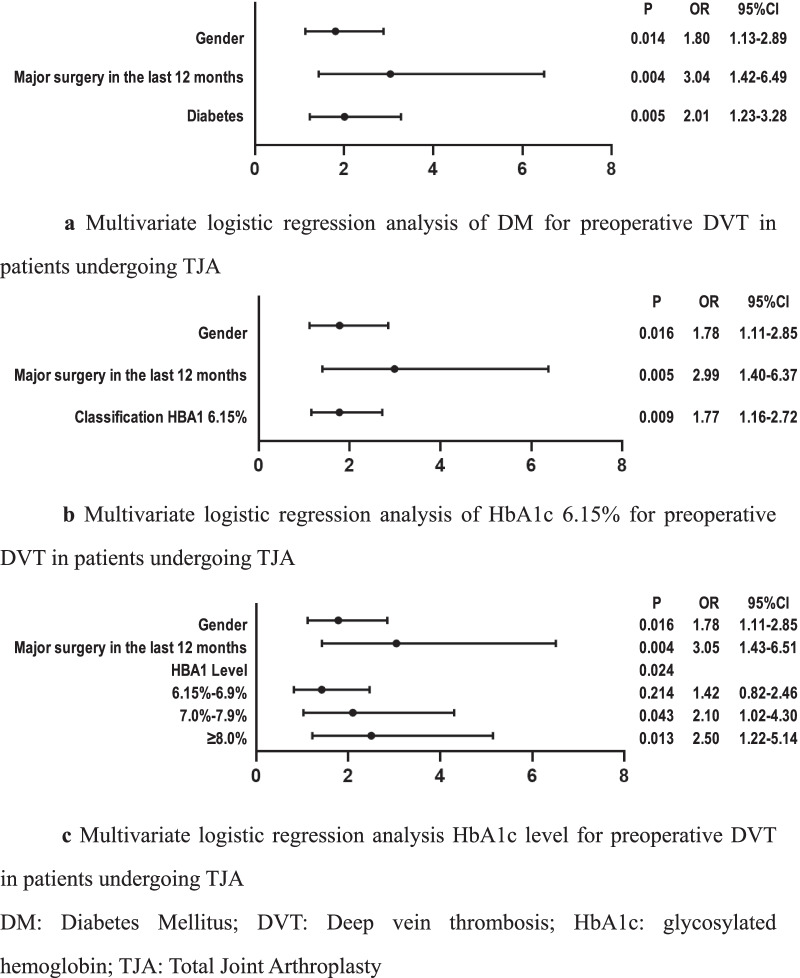
Multivariate logistic regression analysis of DM and HbA1c for preoperative DVT in patients undergoing TJA. **a** Multivariate logistic regression analysis of DM for preoperative DVT in patients undergoing TJA. **b** Multivariate logistic regression analysis of HbA1c 6.15% for preoperative DVT in patients undergoing TJA. **c** Multivariate logistic regression analysis HbA1c level for preoperative DVT in patients undergoing TJA. *DM* Diabetes mellitus; *DVT* deep vein thrombosis; *HbA1c* glycosylated hemoglobin; *TJA* total joint arthroplasty

Considering the multicollinearity of classification of HbA1c at 6.15%, grade of HbA1c, and DM, we conducted a binary logistic regression analysis on these variables separately with gender and the history of major surgery in the last 12 months (Fig. 3). And multivariate logistic regression revealed that the risk of preoperative DVT in TJA patients with HbA1c ≥ 6.15%, HbA1c between 7 and 7.9%, HbA1c ≥ 8%, DM patients, female patients, and patients who underwent major surgery in the last 12 months increased by 1.77 (*P* = 0.009, 95% CI [1.16–2.72]); 2.10 (*P* = 0.043, 95% CI [1.02–4.30]); 2.50 (*P* = 0.013, 95% CI [1.22–5.14]); 2.01 (*P* = 0.005, 95% CI [1.23–3.28]); 1.80 (*P* = 0.014, 95% CI [1.13–2.89]); and 3.04 times (*P* = 0.004, 95% CI [1.42–6.49]), respectively.

## Discussion

Jiao X et al. proposed that the incidence of DVT in early stage following TKA was closely associated with DM and proportional to HbA1c levels [11]. However, their findings were based on a small sample size (224 cases) and only 42 cases were with preoperative DVT. Xiong X et al. reported a 6.78-fold increase in the risk of preoperative DVT in patients undergoing TKA with DM [12]. However, their study also had a small sample size with only 40 cases who had preoperative DVT. In addition, only DM was found to be a risk factor in Xiong X et al.’s study, while other factors such as HbA1c were not examined. The present study is the first one that investigates the association between HbA1c and preoperative DVT in patients undergoing TJA.

### HbA1c

HbA1c reflects the average plasma glucose concentration over a period and is recommended for blood glucose control in those who have DM [11]. Due to the ability to draw the laboratory without fasting, most patients have a high tolerance for HbA1c testing. The relationship between HbA1c and complications has been proven to linearly increase in TJA [14]. Jämsen E found that the elevated preoperative HbA1c level was a risk factor for postoperative hyperglycemia in non-DM patients [19]. Jiao X et al. found that for every 1 unit increase of glycosylated hemoglobin, the incidence of DVT increased 2.35 times and that the incidence of DVT in DM patients within 3 days after TKA was significantly higher than that in non-DM patients and was proportional to the concentration of HbA1c [11]. The results of the present study, similar to those of the research by Jiao X et al., show a 2.1-fold increase in the risk of preoperative DVT with the HbA1c levels between 7 and 7.9%, a 2.5-fold increase in the risk of preoperative DVT with the HbA1c ≥ 8%, and that the higher the HbA1c levels, the greater risk for preoperative DVT.

Nagashima M et al. proposed the cutoff value of preoperative HbA1c of 5.9% and suggested that patients with an HbA1c ≥ 5.9% before TKA may require careful perioperative glycemic control [20]. Stryker LS et al. reported that patients with a preoperative HbA1c level of > 6.7% were at increased risk for wound complications following TJA [21]. Chrastil J et al. reported that patients with DM who underwent TJA with an HbA1c of ≥ 7% had an increased risk of death within two years after the surgical procedure compared with those with an HbA1c of < 7% [22]. Bell EJ et al. found that diagnosed DM and high HbA1c levels tended to be associated with higher VTE risk [23]. Lerstad G et al. observed a tendency for increased risk of provoked VTE in subjects with HbA1c levels of ≥ 6.5%, suggesting that hyperglycemia may predispose to VTE through associated hospitalization or comorbidities [16]. Our study is the first one that has found 6.15% as the cutoff value of HbA1c for the incidence of DVT before TJA. HbA1c ≥ 6.15% is an independent risk factor for DVT before TJA and the risk of preoperative DVT is 1.77 times higher.

Capozzi JD et al. reported that one-third of patients undergoing TJA had undiagnosed dysglycemia detected on routine screening [24]. Three hundred and one patients enrolled in the present study had diagnosed or undiagnosed DM, with a total incidence of 21.7%. Among them, undiagnosed DM patients were 104 cases (41.34%), and the incidence of prediabetes was 41.34%. This finding is consistent with those of the research by Shohat N et al., which showed that the incidence of DM in patients undergoing TJA was 20.6% (diagnosed DM, 59.1%; undiagnosed DM, 40.9%; diagnosed prediabetes, 38.3%) [25]. Marchant MH et al. observed that patients with uncontrolled DM were associated with a significantly increased risk of surgical site infections and mortality following TJA, with a threefold increased risk of stroke, a threefold increase in mortality, and a twofold risk of wound infection compared to controlled DM patients [26]. Shohat N et al. strongly suggest all patients undergoing selective TJA receiving blood glucose screening [25]. Identifying patients with DM and prediabetes could highlight high-risk patients who may benefit from postoperative glucose monitoring and tighter glycemic control [25]. Therefore, we recommend TJA patients who had a higher level of HbA1c be screened for DVT before surgery.

### Hyperglycemia and preoperative DVT

Mraovic B et al. reported a 3.2-fold increase in the risk of PE among patients with DM compared with patients without DM [27]. Jiao X et al. observed that when DM patients have poor glucose level control, the risk of DVT after surgery will increase sharply [11]. Zhang J et al. found that DM was considered a risk factor for DVT and PE in patients undergoing TJA [28]. Jiao X et al. reported a 4.50-fold increase in the risk of preoperative DVT in DM patients undergoing TKA [11]. Xiong X et al. reported a 6.78-fold increase in the risk of preoperative DVT in DM patients undergoing TKA [12]. Our study found a 2.03-fold increase in the risk of preoperative DVT in DM patients undergoing TJA.

Among all patients enrolled in the present study were osteoarthritis (OA). OA and the metabolic syndrome are recognized as the low-grade inflammatory condition with elevations in systemic inflammatory mediators such as interleukin-1 (IL-1), interleukin-6 (IL-6), and tumor necrosis factor (TNF) [29]. A proinflammatory state is very common in DM patients. It has been observed that many clotting factors including fibrinogen, factor V, factor VII, factor VIII, factor X, factor XI, factor XII, kallikrein, and von Willebrand factor are elevated in DM patients [30, 31]; additionally, raised concentrations of other endothelium-derived mediators increase blood viscosity and promote platelet activation and adhesion [32]. Some ex vivo studies documented that spontaneous platelet aggregation is often reported in patients with DM, and this phenomenon correlates with the concentration of HbA1c [33]. DM causes endothelial dysfunction and induces a proinflammatory and procoagulant state, resulting in vascular complications and a shift to a prothrombotic state [34]. Therefore, venous stasis, vascular endothelial dysfunction, and blood hypercoagulability under the combined effect of DM and inflammation may exacerbate the development of DVT in patients undergoing TJA who are preoperatively diagnosed with OA [35]. In patients with bone fractures, DM may lead to stress-induced hyperglycemia, and endothelial cells exposed to elevated levels of blood glucose for an extended period eventually became dysfunctional and are more susceptible to apoptosis in patients who are in a hyperglycemic state [36]. Moreover, DVT can be easily induced because of vascular and endothelial cell injury.

### Other independent risk factors

In this retrospective study, we also observed that female and having undergone major surgery in the last 12 months were also high-risk factors for preoperative DVT in patients undergoing TJA. Female patients had a higher risk of VTE after TKA and THA than male patients, according to Lu Y et al. [37]. Zhang ZH et al. also reported female as a risk factor for VTE after TJA [38]. In our study, female patients had a 1.8 times higher risk of preoperative DVT. Our data showed that the mean BMI of female patients was 25.0 ± 0.12 kg/m^2^ and that of male patients was 23.79 ± 0.15 kg/m^2^, *P* < 0.01. Female patients had higher preoperative BMIs, and the difference was statistically significant. High BMI is often associated with poor hemodynamics and may induce the formation of DVT [38]. History of major surgery in the last 12 months and surgical trauma can also lead to vascular endothelial injury; additionally, significantly decreased mobility during postoperative recovery may cause venous stasis in the lower extremity. Wakabayashi found that prior major surgery is an independent risk factor for DVT after THA [17]. Nauffal D et al. reported that routine and recent surgery was one of the primary causes of death in PE patients [39]. According to Kawai T et al., having undergone major surgery in the last 12 months predisposes patients to increased risk of DVT before THA [40]. Heit et al. found that patients who recently underwent major surgery had a 22 times higher risk of VTE than those who did not [41]. Xiong X et al. reported a 35.37-fold increase in the risk of preoperative DVT in patients who underwent major surgery within 12 months [11]. We found a 3.04-fold increase in the risk of preoperative DVT in patients who underwent major surgery in the last 12 months.

In this study, the correlation with DVT in TJA patients was explored by using materials such as preoperative medical history, preoperative laboratory examinations, and preoperative auxiliary examinations. However, this study has certain limitations. As a retrospective study, some data are incomplete. Moreover, although the critical value of HbA1c with preoperative DVT of 6.15% and the AUC of 0.548 (*P* = 0.006, 95% CI [0.481–0.608]) was statistically significant, future studies with bigger sample size are needed to further verify the critical value.

## Conclusion

Our study found that the independent risk factors for preoperative DVT in patients undergoing TJA were HbA1c ≥ 6.15%, DM, female, and having undergone major surgery in the last 12 months. Patients undergoing TJA who had a higher level of HbA1c should be screened for DVT before surgery because they had an increased risk of preoperative DVT.

## Data Availability

The datasets used and/or analyzed during the current study are available from the corresponding author on reasonable request.

## Abbreviations

AUC: Area under the curve
BMI: Body mass index
CHD: Coronary heart disease
COPD: Chronic obstructive pulmonary disease
DM: Diabetes mellitus
DVT: Deep vein thrombosis
HbA1c: Glycosylated hemoglobin
HB: Hemoglobin
IL-1: Interleukin-1
IL-6: Interleukin-6
IL-17: Interleukin-17
OA: Osteoarthritis
OR: Odds ratio
PE: Pulmonary embolism
ROC: Receiver operating characteristic
TF: Tissue factor
THA: Total hip arthroplasty
TJA: Total joint arthroplasty
TKA: Total knee arthroplasty
TNF: Tumor necrosis factor
PLT: Platelets
VTE: Venous thromboembolism
